# MEET-eaters: An agent-based model of food consumption practices at the household level

**DOI:** 10.1371/journal.pone.0347961

**Published:** 2026-05-22

**Authors:** Christa Blokhuis, J. Gareth Polhill, Marga Ocké, Emely de Vet, Gert Jan Hofstede

**Affiliations:** 1 Chairgroup Consumption and Healthy Lifestyles, Department of Social Sciences, Wageningen University & Research, Wageningen, The Netherlands; 2 Chairgroup Information Technology, Department of Social Sciences, Wageningen University & Research, Wageningen, The Netherlands; 3 Department of Information and Computational Sciences, The James Hutton Institute, Craigiebuckler, Aberdeen, Scotland, United Kingdom; 4 Centre for Prevention, Lifestyle, and Health, National Institute for Public Health and the Environment, Bilthoven, The Netherlands; 5 Chairgroup Global Nutrition, Department of Agrotechnology and Food Sciences, Wageningen University and Research, The Netherlands; 6 Chairgroup Urban Economics, Department of Social Sciences, Wageningen University & Research, Wageningen, The Netherlands; 7 Centre for Applied Risk Management (UARM), North-West University, The Office of the Registrar, Potchefstroom, South Africa; Lusofona University of Humanities and Technologies: Universidade Lusofona de Humanidades e Tecnologias, PORTUGAL

## Abstract

The shift towards less meat and more plant-based consumption is vital for environmental quality, public health, animal welfare, and food security. So far, no studies have examined both the two-way interaction between supermarket supply and household demand, and the role of decision-making about meals within households. This study introduces the agent-based model MEET-eaters, a computational model representing supermarkets, households, and consumers, that was developed to explore these two-way interactions in the Dutch socio-cultural context. The model simulates the effect of supply that is unlimited, fixed, or responsive to the meal choices of households. The approaches to choosing meals are based on the Dutch context. Model development and assumptions were reviewed with experts to enhance model acceptance and understanding. Two interventions were tested, that aimed at either consumer meal preference or supermarket supply. Results show that supermarket supply and household demand both strongly influence the meal choice. Moreover, patterns in meal choices of households are similar under unlimited and responsive supply of supermarkets. The intervention aiming to change supply was most effective when animal-based options were restricted. Results from the intervention changing dietary preference shows that targeting either a large group at random or a smaller high-status group resulted in a similar reduction of animal-based and increase in plant-based meals. For shorter intervention durations, meal choice reverted to pre-intervention patterns once the interventions were discontinued. The results imply that the persistent high availability of animal-based (meat, fish, dairy) options under responsive supply weakens incentives for consumers to purchase plant-based options. In addition, targeting population groups based on status or size could result in more plant-based consumption. Overall, the findings suggest that long-term policies are required to achieve and sustain a shift towards more plant-based diets.

## Introduction

There is increasing recognition in Western countries that a dietary shift from primarily animal-based to more plant-based foods is essential for both human and planetary health [1–6]. Dietary patterns are increasingly understood as emergent outcomes of a complex system [7–9]. The product ranges of supermarkets and consumer demand interact reciprocally within the food environment: “the physical, economic, political and socio-cultural context in which consumers engage with the food system to make their decisions about acquiring, preparing and consuming food” [10]. We do however lack a clear understanding of the two-way interaction between supermarket supply and household demand in understanding the prevalence of meat consumption [7,11–13].

Within households, each of its members influence meat reduction practices, resulting in compromises in the frequency of meat consumption and types of meals consumed [14]. As such, an individual’s social environment provides peer pressure and a source of social modelling for consuming particular foods [15,16]. Shifting dietary patterns towards more plant-based consumption thus requires considering the socio-cultural components of mealtime decision-making in households. Choosing a meal and sharing the meal’s experience within the household occur through culturally-dependent social processes that have so far not been studied in unison with supply-demand interactions of protein sources.

Studying the prevalence of current dietary patterns requires an approach that captures both interactions of consumers with the food environment and the meal decision-making in households. Agent-based modelling (ABM) offers such an approach, as it is suited for simulating social interactions among human individuals (e.g., consumers or supermarket managers) and/or collectives (e.g., households or dinner parties) [17]. Previous studies have aimed to clarify the effect of social influence on meat consumption. However, neither of these studies accounted for the two-way interactions between supermarkets [18–22].

This study aimed to explore the contribution of supermarket supply and household demand to dietary patterns of animal- and plant-based protein sources. In particular, this study aimed to investigate the effect of 1) supermarket responsiveness to household demand in a supply-demand interaction, 2) different culturally-dependent social processes on the meal choice and 3) interventions targeting dietary preferences (i.e., meat-eater, pescatarian, vegetarian, vegan) or supermarket supply on dietary protein choices (i.e., meat, fish, dairy & eggs, plant-based proteins). To explore these effects, MEET-eaters is developed, an agent-based model representing supermarkets, households, and consumers in the Dutch context.

## Background

Eating as a social activity plays a central role in the lives of all people, with most meals being shared with others. The evening meal plays a significant role in family life and involves negotiations between family members about time, food preferences and responsibility [23]. Negotiating about what to eat is a social process that revolves around what is considered normal to eat and the values and practices that are culturally accepted to negotiate meal choice, as well as sharing the meal’s experience.

Culture is defined by a set of shared values and practices that are reinforced through everyday interactions between people [24]. Among the practices that constitute culture, “enjoying a meal together” is a common social practice [25]. In social practice theory, a social practice consists of three “elements”: the material, meanings and competences that comprise an activity (e.g., having dinner) [26]. Each instant of ‘performing’ the social practice offers an opportunity to shift towards more plant-based diets as a result of one of the practice’s elements being changed. For example, a supermarket supply that emphasizes plant-based protein options could change the material element of “enjoying a meal together” towards more plant-based meals. As each element of the practice gradually changes over time, it may or may not transition into a new practice [26].

Social status differences and culture are two aspects influencing the meal choice among household members. Social status is gained by individuals through attention, respect and regard. According to status-power theory [27], status is a concept that illustrates the generic driving forces of social interaction and resulting behaviour, as each human individual strives for gaining status. Changes in status occur through voluntary exchanges. Sharing a meal is in fact one of the practices through which people confirm their status.

The political-economic inclination of a country plays a role in the type of policies that are explored and/or implemented to shift towards more plant-based diets [13]. In the Netherlands, behavioural interventions in food retail aiming to encourage more sustainable and/or healthier food choices mainly consist of extending the existing product range with new or alternative options (e.g., vegetarian meat alternative). In contrast, interventions removing the less sustainable and/or unhealthy choices (e.g., processed meat) are far less common. This approach results from the dominance of neoliberalism, as governmental regulation to protect consumers against less sustainable or unhealthy foods is at odds with the neoliberal paradigm that asserts food choice is a matter of personal responsibility [28].

The Netherlands provides a suitable case to study meal choice among household members as eating together still is a central social practice. Dutch people primarily have dinner with their household members (79%). For both single and multiple-person households, the vast majority of dinners are home-cooked [29]. Portions of meat and fish, or their vegetarian and vegan alternatives in the Netherlands are largest in size during dinner time [30]. As the authors aimed to concentrate on the major contributors of consumption, the model solely simulates dinner. In addition, the Netherlands is a country with a strong tradition of meat consumption and production. A majority of the country’s population eats meat (95,1%) while 1.7% abstains from meat but still eats fish. Others follow a vegetarian (2.6%) or vegan (0.4%) diet [31]. In assessing the environmental and nutritional trade-offs of reduced meat intake for the Dutch population, Zhu *et al.* [32] reported reductions in greenhouse gas emissions and land use, but an increase in water use. The 50% plant-based protein scenario would maintain adequate nutrient intake, only risking vitamin B6 deficiency in women. Another study found that vegetarians and flexitarians in the Netherlands have a significantly lower protein intake and muscle mass than non-vegetarians, but more research is required to determine whether these differences are clinically relevant [33]. According to the most recent data, both consumption and market share of plant-based proteins amount to 40% [31,34]. Supermarkets in the Netherlands collectively agreed to strive for a 60% market share of plant-based protein sources by 2030 [34]. In a letter by the minister of agriculture, fisheries and nature at the time, the Dutch government expressed ambition to transition from 40% to 50% plant-based intake by 2030 [35].

## Methods

### Model overview

MEET-eaters is an agent-based model that represents the two-way interaction between supermarket supply and household demand as elements of a social practice. The model design has been based on several theories and concepts from the social sciences: the power-status theory by Theodore Kemper [27] and social practice theory [26]. Two key motivations guided the development of this model. The first is to provide researchers with insights into how meal choice processes in the household, as well as the physical and social contexts of the food environment, shape dietary patterns. The second is to allow policymakers to explore the effect of a common intervention (changing dietary preference) and a less common intervention (changing supermarket supply) on dietary patterns.

The model’s design is based on several assumptions. The most prominent assumption is that people and their environment continuously influence each other. For MEET-eaters this means that each person-agent is a product of their physical and social environment and is part of the environment of all other agents (persons, households, and supermarkets). Moreover, the physical and social environment are dependent on the people who shape it, for instance, supermarket supply replies to household demand. In this regard, dietary patterns in MEET-eaters are considered a result of the interplay between individual agency and social structures [26,36]. Along the same line it is assumed that consumer behaviour is dependent on context. More specifically, demand for certain protein sources originates from the material, meaningful and competency building blocks that make up consumption practices. Supply of protein sources responds to the demand, driven by the supermarket’s desire for economic profits. Social interaction in MEET-eaters is driven by social status. The meal choice therefore represents an act of balancing each household member’s status demand. The MEET-eaters model enables the exploration of interventions aligned with the Dutch political-economic context, such as changing an individual dietary preference, as well as a less commonly examined intervention (e.g., changing supermarket supply). Finally, distance to a supermarket and availability of protein sources do not limit purchases in MEET-eaters, as the Netherlands is an affluent country with plenty of supermarkets.

The structure of MEET-eaters is mounted on two system archetypes – common behaviour patterns that aid in diagnosing system structures underlying observable events – of the food environment [13]. First, the ‘Supply-demand’ archetype indicates the two-way interaction between supermarkets supply and household demand for the various protein sources. Second, the ‘Success to the successful’ archetype describes why supermarket managers favour meat because it sells better than its alternatives, which suits well with the emphasis of Dutch food retail on profit and the fact they operate in a free market economy. In the paragraphs below, the principal features of the model are briefly described. For full details of the model description and implementation, the authors refer the reader to the ODD + D protocol [37]. The model is available for use or extension by others and is, together with the ODD + D protocol, available from a GitHub repository [38]. All model output data used for analyses is available from an Open Science Repository [39].

### Simulation process

**Fig 1:**
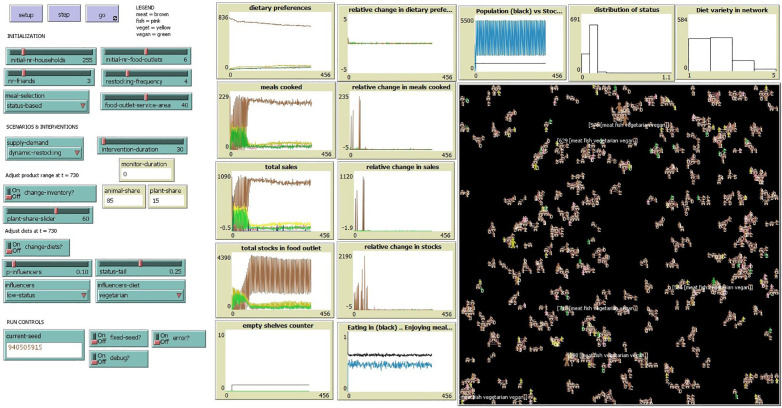
Screenshot of MEET-eaters. The square with a black background shows the representation in the model of the artificial neighbourhood where the simulated people live.

The MEET-eaters model simulates an artificial Dutch neighbourhood including the agents persons, households and supermarkets (Fig 1). Each model run begins by setting up the model (i.e., initializing): creating households, persons, and supermarkets with their characteristic variables. In each household, one or more people reside. Each person is attributed with characteristics such as social status, dietary preference, and social relations. Social status of persons is attributed randomly and not associated with gender or age; there was, moreover, no distinction made between adults and children. Dietary preferences (meat, pescatarian, vegetarian, vegan) are attributed randomly across the artificial population of people. All people are connected in a family network, consisting of their household members, and a friends network, consisting of friends, colleagues and acquaintances. Since the simulation period is ten years, population birth, death, and changes in household composition are omitted, as well as the construction or demolition of houses. Supermarkets are attributed with characteristics such as their inclination to sell sustainable products (i.e., business orientation) and keep track of their inventory and sales. Supermarkets maintain a total inventory based on the number of persons living in their vicinity. Global parameters are used to set up all agents. Some of these global parameters can be adjusted by the user, for example to specify the number of supermarkets. Data sources, value ranges and default values of global parameters are presented in Table 1. All variables characterizing persons, households and supermarkets, and technical details about the model initialization, are reported in the ODD + D protocol [38].

**Table 1:** Data sources, value ranges and default values of global parameters in MEET-eaters.

Global model parameters	Reported values	Value range in model	Default value
Number of households in a neighbourhood [40].	Md = 315 M = 575 P10 = 15 P90 = 1.425	[15,1425]	250
Number of large supermarkets within 1, 3, and 5 km [41].	M = 2 M = 11 M = 23	[1,11]*	6
Household size [42].	M = 2.1	Poisson(λ = 2.1)	Poisson(λ = 2.1)
Dietary identities [31].	Meat-eater: 95.1% Pescatarian: 1.7% Vegetarian: 2.6% Vegan: 0.4%	Meat-eater: [0,1]** Pescatarian: [0,1]** Vegetarian: [0,1]** Vegan: [0,1]**	As value range
Number of friends [43].	5 close friends 10 friends in ‘outer circle’	[0-15]	3
Threshold low or high status.	This is an abstract value between 0 and 1.	Low status: < 0.25 High status: > 0.75	
Inventory levels of animal- and plant-based foods [34].	Plant protein volume: 35–53%	Plant protein volume: [0.35,0.53] Animal plant volume: [1 - Plant protein volume]	As value range
Business orientation.	This is an abstract value of supermarket-agents to add variation in the supermarket’s managerial decisions regarding sustainable product ranges.	[0,2]	As value range
Service radius of supermarkets.	This is an abstract spatial value	[20,60] ***	40
Potential customers per supermarket.	Modern supermarkets count their customers.	[0,n]****	As value range
Safety margin of inventory levels [44].	10%	10%	
Length of inventory replenishment cycle [44].	Sales of specific days (e.g., Mondays) are averaged. Replenishment in Dutch supermarkets occurs once or twice a week.	[1,12]	4

*Total number of supermarkets. **At initialization as reported values. ***Values refer to number of grid cells in the modelled space. The entire model space consists of 61x61 grid cells. *****Count of persons within the service radius.

After initialization, the model runs for ten consecutive years of 365 days, each time step representing one day. The duration of one model run thus equals 3650 time steps. For each timestep, a series of procedures is performed. Every night, people have dinner together. Before dinner, at most one household member is selected as the cook, who invites family members and, occasionally, friends to a dinner party. The cook then discusses the meal choice with those attending the dinner party and purchases this protein source at the supermarket. If the chosen protein source (e.g., fish) is not available, the cook will either choose an alternative (e.g., meat, vegetarian, or vegan) or visit another supermarket. Supermarkets adjust their inventory levels of meat, fish, vegetarian and vegan based on sales and requests by customers. Details of each of the modelled procedures can be found in the ODD + D protocol [38].

During each run, users of the model can view several graphical and numerical outputs in the model interface (Fig 1), including the distribution of dietary preferences in the population of people, how many people eat at home or at a friend’s place, and the total sales per protein source. The combined outputs of inventory levels and type of meals cooked reflect the composition of the dinner practice, i.e., the supermarket supply and household demand. Dietary preference is monitored as people do not always consume their preference and allows detection of any differences between preferences and actual consumption. Dietary diversity is a metric showing the number of different dietary preferences within each person’s social network and indicates the degree to which the network is homogeneous or heterogeneous.

### Model development, verification and validation

The model’s design and implementation were endorsed using sensitivity analysis and model validation. A sprint planning approach was applied to develop the model in a modular and iterative fashion. First, the model was designed, i.e., agreements settled about its assumptions and scope, in collaboration with the entire team using class diagrams (listing the agents and their characteristics), relationship diagrams (showing how each agent relates to the other agents) and flow charts (visualization of decisions made by the agents). Second, the features of the model design were outlined for each sprint and completed before progressing to the next sprint. In developing the model, each agent decision-making process was added as a separate module to enable transparency and easy adjustment. Any problems encountered in implementation were discussed and, if necessary, the model design was adjusted. A list of the sprints, a log book, and an error log were kept to document model development. With each sprint, the model was expanded with additional features or refined with further details. For instance, when introducing the module for getting groceries (see submodels in ODD + D protocol), cook-agents would repeatedly purchase groceries and exhaust the supermarket’s supply. This issue was resolved by introducing stricter conditions to ensure that cook-agents obtained groceries only if they had not already done so. After completing the procedure for obtaining groceries, a separate procedure was introduced for obtaining alternative protein sources, allowing users of the model to easily adjust the conditions under which cook-agents choose alternatives to their preferred protein source. Lastly, the model was thoroughly tested during and after model completion and a code check was conducted by an experienced agent-based modeller from outside the author team.

In order to explore the behaviour of the MEET-eaters model, default conditions were explored. Baseline model results were established by running the model under default conditions (Table 1), providing a reference point for assessing intervention effects. Default conditions were ran 100 times and model outputs were recorded for each time step. Homeostasis, the point when the model reaches a stable state after initialization, is defined as the stabilization of the variation in the sum of interquartile ranges and the median (MD ± IQR) of model outcomes over time.

A sensitivity analysis was performed to determine the impact of each parameter on model outcomes. Following the suggestion by Broeke *et al.* [45], we applied One Factor at A Time (OFAT) in combination with Partial Rank Correlation Coefficient (PRCC). OFAT helped us to determine the impact of each global and agent parameter on the model outcomes ‘Types of meals cooked’ and ’Percentage of persons enjoying dinner’, and decide upon the default values for the intervention runs. Selected settings for OFAT are presented in Table 2. PRCC is a global sensitivity analysis methods that works best for variables that are linearly related and when the controlling variables are not highly correlated, which we observed for the results from the OFAT sensitivity analysis. Another reason that PRCC was an appropriate choice pertains to the number of outliers that was observed in the plots from the OFAT sensitivity analysis. PRCC was performed on three global parameters: number of supermarkets, number of friends, and the length of the replenishment cycle. The PRCC function is provided in equation (1). Y is the output variable, either a ‘Type of meals cooked’ or ‘Inventory levels’ for one of the four protein sources; X is the variable for which the relationship with Y is tested. A and B represent the controlling variables. If the number of supermarkets is X, then the other two variables are the controlling variables.

**Table 2:** Table with settings for OFAT sensitivity analysis.

parameter	type	values
Initial-nr-food-outlets	factor	1,5,10
Initial-nr-households	factor	25, 250,750,1500
Nr-friends	factor	0,3,6
Restocking-frequency	factor	1,4,7
Food-outlet-service-area	factor	20,40,60
Supermarket visits	factor	1,2,3,4,5,6,7,8
Meal variety	Numeric	0.1,0.2,0.3,0.4,0.5
Status	Numeric	0,1
Neophobia level	Numeric	0,0.1,0.2,0.3,0.4,0.5,0.6,0.7,0.8,0.9,1
Cooking skills	Numeric	0,0.2,0.4,0.6,0.8,1

The term with *ρ* represents Spearman’s rank correlation coefficient between X and the residuals of Y from the regression on A and B. For full details of the sensitivity analysis, the authors kindly refer the reader to the document with sensitivity analysis at GitHub [38].


$$\begin{array}{c} PRCC(X,Y|A,B)=\rho_{xy,ab} \end{array}$$ (1)

Model validation was applied to ensure MEET-eaters’ design sufficiently represents the reality of supply-demand interactions between supermarkets and households in the Dutch context. The model was qualitatively validated using group meetings with experts in food consumption behaviour (who were inexperienced in modelling) and separate meetings with modelling experts at several stages of the model design and implementation process. The experts were recruited through the network of the author team and meetings were conducted in-person or online. A first design of the model was presented to a group of experts on food consumption behaviour who were mainly non-modellers (Table 3 participants 1–9). Their feedback resulted in several adjustments and refinements of the model design. These included selecting social practice theory as the main theoretical framework; broadening the concept of social modelling to social influence; refining the flowchart for person-agents eating alone. In addition, we clarified that the supermarket’s supply reflect managerial decision-making, that model outcomes reflect elements of a social practice rather than consumption only (i.e., supermarket supply *and* household demand), and that the physical food environment (i.e., the supermarket supply) results from the interaction with consumers.

**Table 3:** Overview of consulted experts.

Participant ID	Area of expertise	(Non-)modeller
1	Public health and nutrition	Non-modeller
2	Consumption sociology	Non-modeller
3	Epidemiology, nutrition and health	Non-modeller
4	Behavioural change	Non-modeller
5	Clinical nutrition and health	Modeller
6	Consumer behaviour and societal transition	Non-modeller
7	Food environment policy	Non-modeller
8	Industrial design engineering	Non-modeller
9	Human geography	Non-modeller
10	Modelling social systems	Modeller
11	Social simulation and network analysis	Modeller
12	Sociology	Non-modeller
13	Operations research	Modeller
14	Inventory management	Modeller

A first version of the simulation model was presented to simulation experts (Table 3, participants 10 and 11), who provided advice on implementing interaction within and between households. In presenting the first version of the simulation model to the group of experts (Table 3, participants 1–9), the author team explained how their feedback was processed, for example the choice for social practice theory and a business orientation reflecting managerial choices in supermarket supply (see the ODD + D protocol at Blokhuis and Polhill [38]). Their feedback prompted further refinement of the model’s procedures for selecting the meal, obtaining groceries, and evaluating the meal. Later, a meeting with an expert in social practice theory (Table 3, participants 12) was held to refine the implementation of this theory in MEET-eaters. In addition, two experts on supermarket logistics (Table 3, participants 13 and 14) were consulted to implement a more realistic inventory management process in MEET-eaters.

### Model setups

One of the merits of simulation models is the possibility to study situations that are hard to study in reality, for example because their core dynamics have not been (fully) formalized or available data do not support a systemic conceptualization [46]. The two-way interaction between supermarket supply and household demand is an example of such a situation. Dutch supermarkets are unlikely to participate in an experiment that might compromise their profit, and there is a lack of data on meal-time decision-making from the perspective of the power-status theory by Kemper [27]. Therefore, the MEET-eaters model is developed to explore the respective contribution of supermarket supply (i.e., the physical environment) and decision-making about meals within households (i.e., the social environment) to the consumption practice of having dinner. The MEET-eaters model contains three supply-demand setups to address the effect of supermarket responsiveness to household demand and dietary preferences (Table 4). The supermarkets and households are treated as subsystems that are partially or fully linked to explore the respective effect on the model’s outcomes, an approach to study complexity across (sub-)models put forward by Parker *et al.* [47]. First, the infinite-supply setup eliminates the role of supermarkets in influencing household demand and dietary patterns and allows for observation of only the social environment on type of meals cooked. Second, the static-supply setup fixes supermarket supply, assuming that supermarkets are not responsive to household demand. This reflects a situation in which there is affluent abundance of food but supermarket managers are not incentivized by making profit. Finally, the dynamic-supply setup reflects the reality of interaction between supply and demand in a free market economy. Prior to testing these supply-demand settings, we hypothesized that the dynamic-supply setup would result in the largest preference and consumption of meat, as well as the largest inventory levels of meat (compared to static supply).

**Table 4:** Initial model setups: supply-demand options.

Setup	Rationale	Setting in interface	Values
**[Fig in Table 4 infinite inventory.jpg]**	With this setup the model allows for exploring the effect of household decision-making on type of meals cooked; food product availability is infinite.	SUPPLY-DEMAND	INFINITE-INVENTORY
**[Fig in Table 4 static inventory.jpg]**	With this setup the model allows for exploring the effect of the interaction between food product availability and household decision-making on type of meals cooked, while the food outlets are not responsive to sales.	SUPPLY-DEMAND	STATIC-INVENTORY
**[Fig in Table 4 dynamic inventory.jpg]**	With this setup the model allows for exploring the reciprocity of food product availability and household decision-making on type of meals cooked, while the food outlets are responsive to sales.	SUPPLY-DEMAND	DYNAMIC-INVENTORY

Cultural context plays a crucial role in determining whose dietary preference within a household are prioritized, and the resulting decision impacts future social relationships through status conferrals, for example by showing respect for a well-cooked meal. The MEET-eaters model includes culturally-dependent meal choice processes that are typical for the Netherlands. As a result of the strong feminine nature of its society [48], Dutch people would rather use the status dimension to settle a conflict (e.g., finding an excuse for not joining dinner) then openly wield power (e.g., by refusing to eat something). Any negotiations among household members about which protein source to eat for dinner is hence likely settled by status (the preference of the individual with the highest social status) or the majority (the preference of the majority). This study diverged from a commonly-used approach to execute (food) decision-making called the “follow-the-average” (FTA) strategy [49]. Models implementing FTA assume 1) that the majority is decisive and 2) that a vegetarian will default to vegetarian options (e.g., Scalco *et al.* [19]). In the MEET-eaters model, the majority strategy aligns with the first assumption of the FTA, but we diverged from the second assumption as findings from literature provide contradictory evidence. For example, Rosenfeld and Tomiyama [50] reported that vegetarians sometimes violate their diet in an attempt to harmonize social situations. The MEET-eaters model contains three setups for meal selection to explore the effect of decision-making about meals within households on supermarket supply, household demand and dietary preferences (Table 5). First, random meal-selection serves as the default to which the other two meal choice processes are compared. With this option, the cook will randomly select one of the meal options: meat, fish, dairy & eggs, or plant-based. Second, with status-based meal-selection, the cook will select a meal based on the dietary preference of the guest with the highest status, including themselves. It is implicitly assumed in MEET-eaters that the most status-worthy dinner guest deserves to choose the meal. Finally, in the majority-based option the cook will select the modal meal preference of the dinner guests. As a large majority of the simulated population prefers meat, we hypothesized that a status-based meal-selection would result in the largest consumption of meat.

**Table 5:** Initial model setups: meal-selection options.

Setup	Rationale	Setting in interface	Values
**[Fig in Table 5 random.jpg]**	With this setup the cook will randomly select one of the meal options: meat, fish, dairy & eggs, or plant-based.	MEAL-SELECTION	RANDOM
**[Fig in Table 5 status-based.jpg]**	With this setup the cook will select a meal based on the preference of guest with the highest status, including himself.	MEAL-SELECTION	STATUS-BASED
**[Fig in Table 5 majority.jpg]**	With this setup the cook will select the most preferred meal preference of the dinner guests. In case of a tie, the cook will randomly select one of the two remaining options.	MEAL-SELECTION	MAJORITY

### Model interventions

The MEET-eaters model includes two interventions, that aimed at either dietary preference of person-agents or supply of supermarket-agents Table 6. Literature suggests that interventions targeting nutrition outcomes can improve short-term dietary behaviours, yet evidence for sustained dietary change is limited [51]. In addition, Escaron *et al.* [52] reported that from 33 interventions that promoted healthy food choices in supermarkets, the average duration amounted to 7 months (M = 4, SD = 9.3) and less than half of the interventions (n = 15) yielded a significant effect post-intervention. To explore any tipping point toward more plant-based cooking, for both interventions tested in this study several durations are explored. We hypothesized that the longer an intervention is implemented, the greater the number of plant-based meals and the more enduring its effects, potentially lasting indefinitely. For both interventions, a duration can be set between 1 (i.e., day) and 2920 (i.e., eight years) time steps. An adjustable duration can provide users (e.g., policymakers, researchers) with relevant insights into the effect of the intervention period. Each intervention is implemented at the two-year mark (~730 days) and runs for the selected intervention period. Both interventions were run with supply-demand setup “dynamic-inventory” and meal-selection setup “status-based”, as the authors aimed to test the interventions with a setup most representative for the Dutch situation. We ran each experimental setting 20 times for the full simulation period of ten years, and recorded the model outcomes at the end of each year, resulting in ten data points per run.

**Table 6:** Model interventions.

Intervention	Rationale	Setting in interface	Values
Dietary change **[Fig in Table 6 dietary change.jpg]**	With this target-seeking intervention the model allows for finding out to what extent a dietary change in the population can contribute to the protein transition and how the change depends on the number and type of persons switching diet.	INFLUENCERS	TRUE
		status-influencers	[>0.75, <0.25, Random]
		diet-influencers	[Meat, Fish, Vegetarian, Vegan]
		p-influencers	[0-1]
		duration	[7 −730]
Inventory change **[Fig in Table 6 inventory change.jpg]**	In this target-seeking intervention the model allows for finding out to what extent adjustment of food outlet stocks can accelerate to or hamper the protein transition.	CHANGE-PROTEINS	TRUE
		plant-fraction	[1*−70]
		duration	[7 −730]

*Initial minimum plant fraction is dependent on model initialization but is always smaller than the animal fraction as a result of the initial distribution of dietary preferences, with person-agents preferring a vegan diet, thus plant-based meals, amounting to 0.4% of the population.

For the intervention targeting dietary preference it was assumed that different subgroups in the population, based on their social status and size, have varying influence on the dietary preference of their peers. The rationale of this intervention is informed by several theoretical concepts. First of all, based on the status-power theory by Kemper [27], we assumed that high status individuals are more influential in expanding plant-based cooking compared to low status individuals and groups of individuals with random status. Second, research suggests that social context, particularly the behaviour of those around us, strongly affects individual food choices [16,53]. For these reasons, we assumed that a larger group would exert more influence on plant-based cooking than a smaller one. When combined, we hypothesized that a small group of high status would be most effective in changing dietary preferences. Therefore, the intervention targeting for dietary change features the possibility to investigate the effect of targeting different subgroups in the population. Users can request a fraction of the population (e.g., p = 0.2) with a specific status (e.g., status <0.25), to change their diet towards a user-preferred dietary preference (e.g., “vegan”) for a specified amount of time (e.g., one month). At termination of the dietary change intervention, person-agents that were participating in the intervention can freely adjust their dietary preference again. The mechanisms leading to a change in the population’s dietary preferences (e.g., food safety incidents, educational programs) are outside the scope of this study.

For the intervention targeting supermarket supply it was assumed that a larger supply of plant-based protein sources would lead to an increase in vegan meals. The rationale of this intervention is supported by literature, where it is reported that choice architecture interventions result in a significant change in food purchases [54]. However, it is unclear under what conditions this significant change is attained for the various protein sources (meat, fish, dairy and eggs, plant-based proteins). This intervention explores for which ratios of animal- and plant-based product ranges vegan cooking will reflect the 50:50 consumption ratio expressed by the Dutch government [35]. Among the ratios tested, the goal set by Dutch supermarkets to attain a 40:60 supply ratio of animal- and plant-based protein sources [34] is included. Prior to the experiment, we hypothesized that a significant increase in vegan meals would show after the ratio of animal- vs. plant-based protein sources was set to 50:50. The intervention is implemented by adjusting the inventories according to the user-specified ratio and the model’s supply-demand setting is changed to “static inventory”, to ensure a fixed product range until the end of the intervention period. At termination of the intervention, supply-demand is set back to “dynamic inventory”, the default condition for interaction between supermarkets and households.

## Results

### Baseline model: the model without interventions

**Fig 2:**
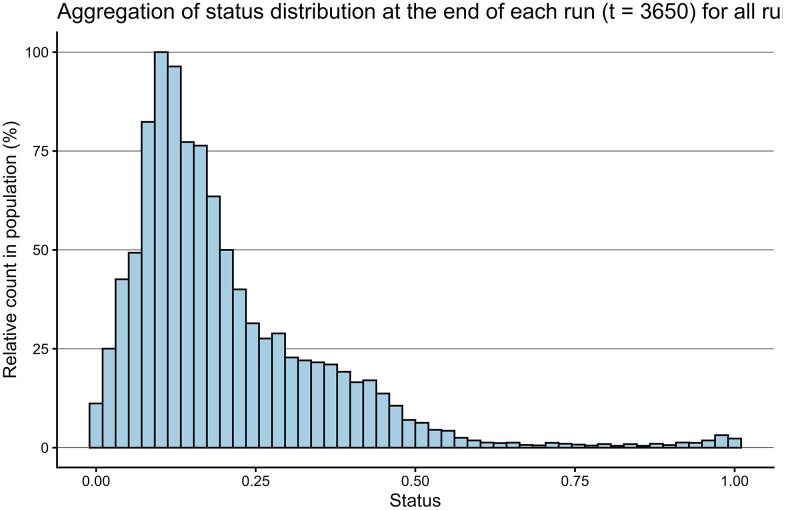
Histogram showing aggregated status distribution at the end of each run under baseline conditions.

Population sizes for all baseline runs showed a bimodal distribution of two bell curves, one situated in the smaller population sizes (n = 5124, M = 773, SD = 23) and another containing larger population sizes (n = 900, M = 1548, SD = 32). Status is normally-distributed at initialization and develops into a right-skewed distribution as shown in Fig 2 reflecting a hierarchy with a majority group of lower status and a minority of higher status.

**Fig 3:**
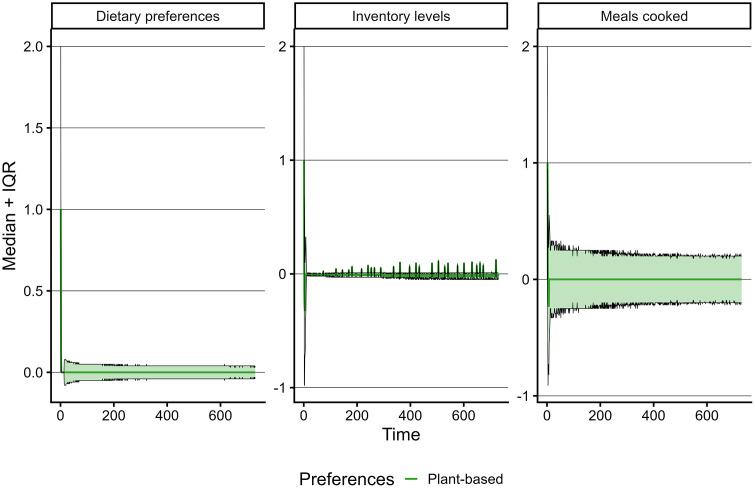
Graphs showing a stabilization of the convergence in the sum of median and interquartile ranges for plant-based model outcomes meals cooked, inventory levels, and dietary preferences. The graphs for meat, fish, and dairy & eggs are available through the Github repository [34].

Trends for meals and dietary preferences are exhibiting homeostasis at different stages in the model run but amply before 730 time steps (i.e., two years) (Fig 3). Trends for relative changes in inventory levels continued to show spikes at irregular intervals throughout the entire run. As these types of patterns are common for supply-demand systems [55], we considered these results typical model behaviour. Therefore, the first two years are considered the time needed by the model to stabilize and experimental conditions were initiated from year two onwards.

**Fig 4:**
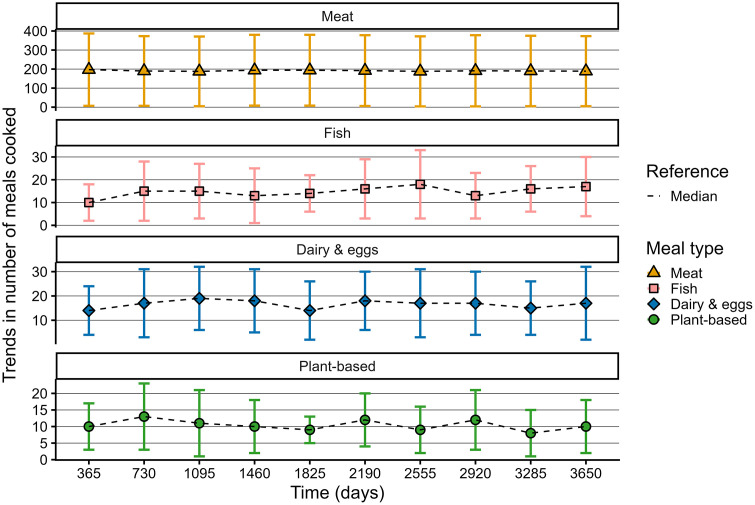
Graphs showing median and interquartile ranges of meals cooked for each meal type at the end of each year under baseline conditions.

**Fig 5:**
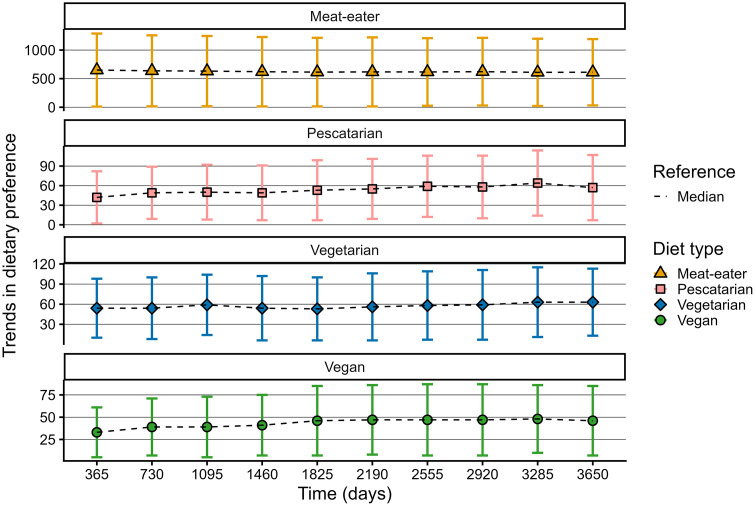
Graphs showing median and interquartile ranges of dietary preferences at the end of each year under baseline conditions.

Meat meals were consumed an order of magnitude more often than the other three meal types (Fig 4); a similar pattern was shown for the products’ inventories. The fact that dairy consumption in the Netherlands, in particular milk, is much higher compared to other countries [56], data for dairy & eggs may overestimate the amount consumed at dinner. Inventory levels under baseline conditions demonstrate an abundance, which resembles the situation of the Netherlands. Median baseline values of total plant share vary only minimally over time, between 3% and 5%, which resembles the market share of plant-based sources such as legumes, nuts, seeds and plant-based dairy- and meat alternatives [34]. Across meal types, dietary preferences, and plant share, interquartile ranges reached up to twice the median, distributions were non-normal, and outcomes varied considerably between runs. Together, these patterns imply a broad spectrum of possible baseline outcomes, which is also typical for a complex system. On average, a slight decrease in meat consumption and slight increases in the consumption of fish, dairy & eggs, and plant-based proteins were observed; similar patterns showed for the development in dietary preferences (Fig 4,5). This trend under baseline conditions indicates a minimal convergence in meals and dietary preferences, which is reported by studies as well [57,58].

### Sensitivity analysis

Based on the outcomes of OFAT (Table 7), the size of the service area and social status of person-agents preferring vegan meals positively influence the choice for plant-based meals, while number of friends and neophobia of person-agents appear to negatively influence this choice. Several parameters, including meal variety (i.e., a fixed variance in the quality of meals cooked that determines meal enjoyment) and cooking skills of person-agents, seem unrelated to plant-based cooking.

**Table 7:** Summary of OFAT-based sensitivity analysis.

Parameter(s)	Model outcome	Relationship with outcome
Initial number of food outlets	Plant-based meals cooked	No trend
Initial number of households	Plant-based meals cooked	Slight negative trend
Number of friends	Plant-based meals cooked	Negative trend
Restocking frequency	Plant-based meals cooked	No trend
Food outlet service area	Plant-based meals cooked	Positive trend
Supermarket visits	Plant-based meals cooked	No trend
Meal variety	Plant-based meals cooked	No trend
Meal variety	Percentage meal enjoyment	No trend
Status of people preferring vegan	Plant-based meals cooked Other meals cooked	Positive trend Negative trend
Neophobia	Plant-based meals cooked	Negative trend
Cooking skills	Plant-based meals cooked Percentage meal enjoyment	No trend Logistic growth (S-curve)
Cooking skills & Meal variety	Percentage meal enjoyment	Logistic growth (S-curve)

**Fig 6:**
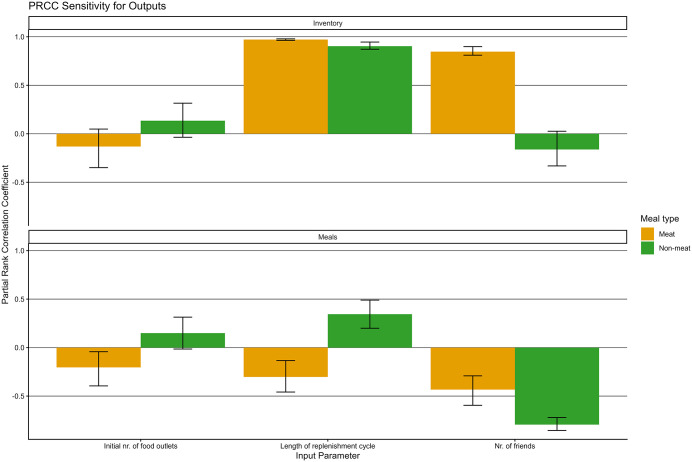
Results for global sensitivity analysis (PRCC) with non-meat (fish, vegetarian, vegan) grouped together.

Although several of the variables did not show a clear linear relationship with model outcomes (see [38]), the high number of outliers and the absence of correlation between variables justified the choice for PRCC. Results of the PRCC for meat and non-meat (fish, vegetarian, vegan) are shown in Fig 6. The opposing directions of meat and non-meat meals and inventory levels stand out. Because the majority of the population prefers meat, the supermarkets reflect this preference in their inventory levels. In particular, the length of the replenishment cycle (i.e., how fast meat alternatives are restocked) and the number of friends (i.e., the likelihood that a high-status meat-preferring person-agents attends the dinner party) appear to be most relevant in determining model outcomes. All details of sensitivity analysis can be found through the GitHub repository [38].

### The respective contribution of supply and demand to dietary patterns

**Fig 7:**
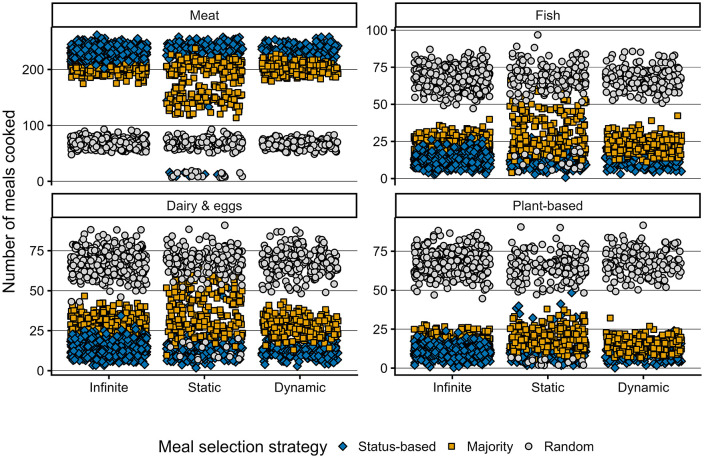
The effect of meal selection strategies on number of meals cooked. Meal selection strategies are marked by shape and colour: status-based is represented by the blue diamonds; majority by orange squares; random by grey circles.

**Fig 8:**
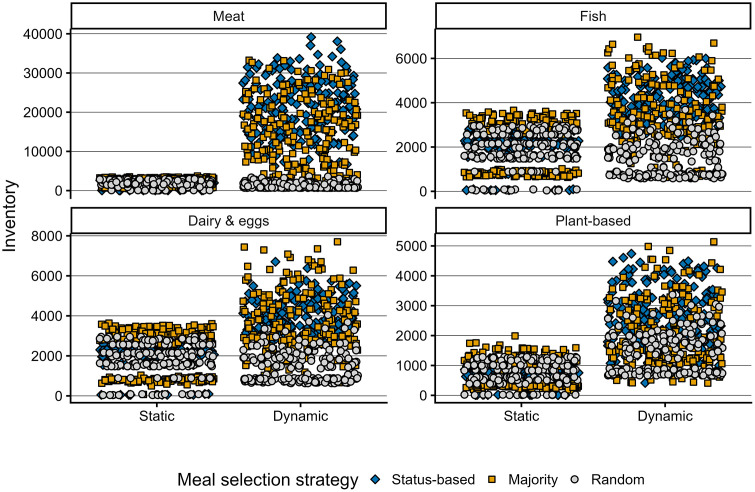
The effect of meal selection strategies on inventory levels (bottom) across supply-demand settings. Meal selection strategies are marked by shape and colour: status-based is represented by the blue diamonds; majority by orange squares; random by grey circles.

The respective contribution of supermarket supply and household demand to dietary patterns was explored in MEET-eaters by varying the initial supply-demand settings between infinite, static, and dynamic inventory (Table 4). The effect of each supply-demand setting on types of meal cooked is demonstrated in Fig 7. Contrary to the hypothesis, most meat meals are cooked when supply is infinite rather than when the supply is responsive. For meals featuring non-meat (fish, dairy and eggs, or plant-based proteins), most of these meals are cooked when supermarkets offer a fixed product range. In line with the hypothesis, results show that median inventory levels of all meal types are largest under the dynamic setting (Fig 8).

### The effect of meal-selection setup on dietary protein choices and inventory levels

Each meal selection setup results in distinct differences in cooking frequency across meal types and inventory levels, implying a significant effect of meal-time negotiation on dietary protein choice (Fig 7 and 8). Results from the random meal selection setup on type of meals cooked differs strongly from the other two setups and appears similar across the three supply-demand settings, indicating a minimal effect Fig 7. Contrary to the hypothesis, the status-based meal-selection setup appears to cause the largest consumption of meat; the majority setup consistently results in more meals featuring fish, dairy and eggs, and plant-based-based proteins. These results suggest that a status-based meal selection conserves initial meat-heavy dietary patterns more strongly than a majority-based selection. The majority-based meal-selection setup, however, shows the largest variety in number of meals cooked under static-supply. The fixed nature of the supply incentivizes cook-agents to make an alternative choice relative to their preferred meal, implying the essential role of supermarkets in dietary patterns. The results furthermore demonstrate that both the status- and majority-based meal selection setups strongly affect inventory levels under dynamic-supply (Fig 8), unlike the random meal selection setup, as purchasing a random meal is less dependent on availability than purchasing a particular meal.

### Experimental conditions

**Fig 9:**
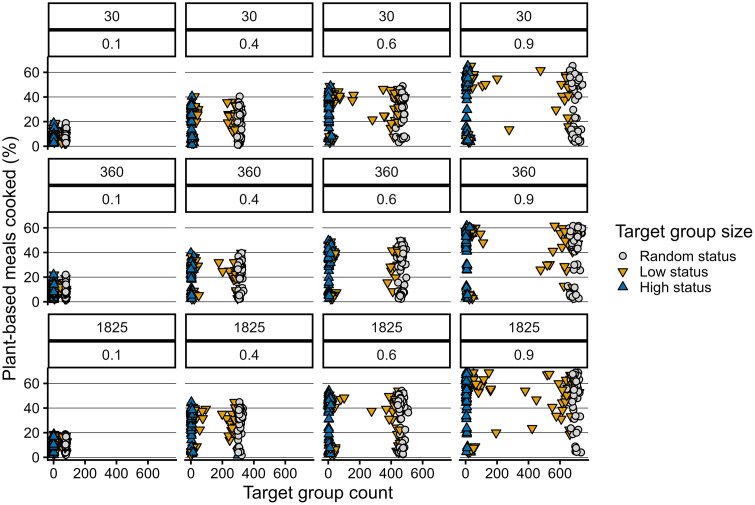
The combined effect of target group (random status, low status, high status) and target group size, intervention durations (30, 360, and 1825 days) and target group fractions 0.1, 0.4, 0.6, and 0.9 on relative number of plant-based meals cooked.

**Fig 10:**
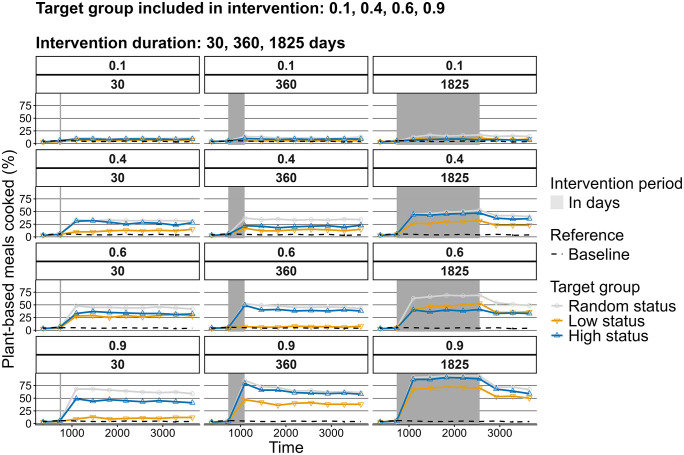
The effect of target group, target group fraction, and intervention duration on median plant-based meals cooked over time.

**Fig 11:**
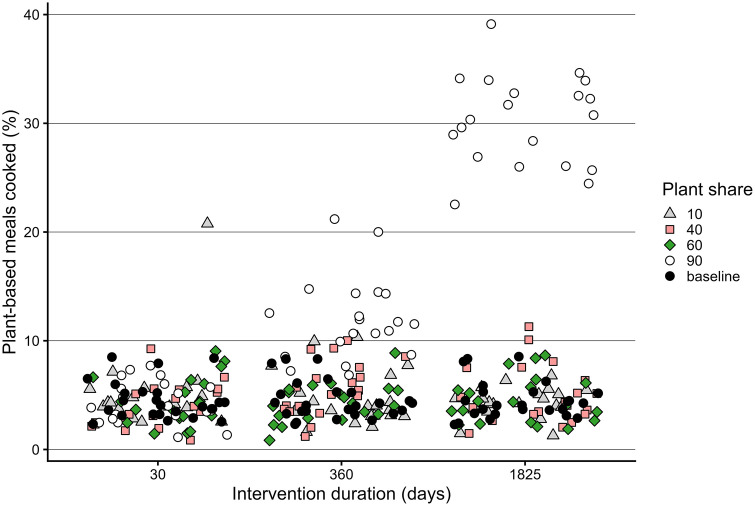
The effect plant-based share in supermarket supply and the duration of the inventory change intervention on relative number of plant-based meals cooked at the end of each experimental run (t = 3650).

**Fig 12:**
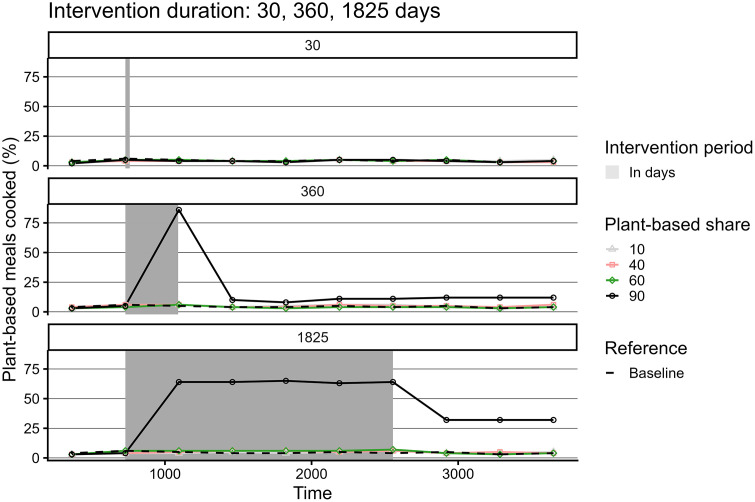
The effect of plant-based share in supermarket supply and intervention duration on plant-based meals cooked over time.

In line with the hypothesis, the intervention duration was positively associated with number of plant-based meals, both while the interventions were active and after termination (Fig 10). However, in all cases, plant-based cooking decreased after an intervention was discontinued and would, in case of the shorter interventions, drop to pre-intervention values. Results from the dietary change interventions partly align with the hypothesis. Group size matters in shifting towards more plant-based diets, yet the type of individuals in the group mediates the exerted influence (Fig 8). We observe a strong effect of large group of randomly selected person-agents on plant-based meals cooked (Fig 9), though intervening in the high-status groups, albeit smaller, exert a comparable influence. (Fig 10). Contrary to the hypothesis, a 50:50 ratio of animal- and plant-based protein sources in supermarkets is not sufficient to significantly increase plant-based meals. What is more, results demonstrate that a 60% plant-based protein share, the goal set by Dutch supermarkets, is also not sufficient to increase plant-based cooking compared to baseline conditions Fig 11. When plant share is set to 90%, the 50% plant-based cooking mark is surpassed, if the intervention lasts at least a year. Nevertheless, this level of plant-based cooking is not sustained after the intervention is discontinued (Fig 12).

## Discussion

This study aimed to explore the contribution of supermarket supply and household demand to dietary patterns of animal- and plant-based protein sources. The MEET-eaters model offered a means to explore the role of supermarket responsiveness in demand, and that of decision-making about meals within households, in types of meals consumed. Two interventions, targeting either dietary preferences or supermarket supply, provided more insight in the respective contribution of product availability and household demand to the dinner practice. Overall, the model outcomes underscore the importance of both supermarket supply and household demand in understanding dietary protein choices.

### The respective contributions of supply and demand

The findings of this study provided insight into the role of supermarket responsiveness in maintaining initial meat-heavy diets and product ranges. In a situation with a strong practice (here: meat for dinner), a product range that is unlimited (infinite inventory) or responsive to actual demand (dynamic inventory), results in a reinforcement of this consumption practice. Model results show that when availability is not responsive (static inventory), supermarkets do not sufficiently supply meat for the practice to remain or expand. Less prominent practices, in this study the consumption of fish, dairy & eggs, and plant-based proteins for dinner, are strengthened in situation with non-responsive supermarkets. Our findings indicate that in case meat availability is abundant, as in both the infinite or dynamic inventory setups, person-agents are not persuaded to choose an alternative. This implies that changes in choice architecture of Dutch supermarkets might only support more plant-based diets when the choice for certain animal-based products is limited. These model outcomes add a novel insight, as to the best of our knowledge, previous studies using social simulation modelling in the context of the protein transition have not included availability or food decision-making in retail [18,19,21,22].

### The effect of decision-making about meals

The results of this model emphasize that assumptions made regarding decision-making about meals within households significantly impact both the type of protein sources consumed as well as the protein sources offered by food outlets. Generally, the significant difference between random and status- or majority-based meal selection strategies that were found in this study aligns with literature stating that dinner time decision-making is driven by certain social and cultural rules [59,60]. In exploring the effect of a status-based meal selection strategy, our model results indicated that high status associates with a meat-based diet, a finding in line with literature [61]. However, based on the assumptions of our model design, this finding is likely a result of the meat-eaters being the largest subgroup in the population and reflects the dominant consumption practice.

### The effect of adjusting dietary preference or supermarket supply

Results of the interventions tested in this study reveal that more plant-based cooking can be attained by person-agents adjusting their dietary preferences and/or food outlets expanding their supply of plant-based products. Experimental outcomes indicate that plant-based cooking relapses after interventions end, irrespective of their duration. Even the longest intervention, spanning five years, shows a relapse in plant-based meals cooked after an intervention has been withdrawn. As these findings demonstrate a lack of acculturation of intervention-induced behavioural patterns, we suggests long-term policies, as well as expanding the modelled period and model intervention duration to explore any tipping points in sustained increases in plant-based cooking. Moreover, our model results underline the need to study the effect of long-term interventions in real-world settings as well as longer-term follow-up measurements, in particular because interventions addressing behaviour change toward less meat and/or more plant-based consumption, mainly consist of single-time interventions, while fewer studies extended up to several weeks or months [62]. Empirical studies investigating the effect of interventions rarely report longer term results, which is also the case for choice architecture interventions [63]. A review by Escaron *et al.* [52] found that for 31 interventions promoting healthy food choices in food retail, 28 lasted less than a year, with a median duration of 4 months.

The relapse in plant-based meals cooked in MEET-eaters after withdrawing the interventions can potentially be attributed to two reasons: the trends in dietary preference, and the inventory levels of each protein source at the start of the intervention. First, person-agents eat different meal types which they might (not) enjoy. Hence, the increment for meal enjoyment, which is 0.01 (see ODD + D protocol), is added to and subtracted from all dietary preferences throughout each run, creating a gradual process for individuals to change their preferences. The MEET-eaters’ design illustrates the resilience of current dietary protein choices as a result of slow-changing preferences. However, although this observation aligns with the literature, a range of other barriers has been identified that explain why consumers do not reduce meat [11,64,65]. Second, the inventory levels for each of the protein sources are relatively high (1000 + items per protein source across multiple food outlets) compared to the population size (~1500), indicating abundance. As a result, all of the tested plant share values still accommodate an animal-based product range sufficient to sustain pre-existing dietary patterns. Our findings partially align with literature. The review by Tirion *et al.* [66] reported that restructuring the physical food environment was one of the most effective interventions in encouraging sustainable food consumption (e.g., choosing veg*n food, reducing meat and fish consumption). However, the authors did not specify in which way the restructuring was implemented, effect sizes varied between very small and very large, and duration of interventions was not mentioned. Completely or partially restricting animal-based products in food outlets, for example a red-meat free campus or Meatless Mondays, was found to be highly effective in reducing meat consumption [67]. However, Kwasny *et al.* [62] reported that meat reduction is stimulated by increasing the variety and visibility of veg*n options and several intervention studies that aimed to increase the visibility of plant-based alternatives found that while plant-based sales often increased, meat sales remained unchanged [68–71]. Both the literature and the outcomes of the MEET-eaters model highlight the need for further research to determine from which point onwards, and under what conditions, plant-based cooking will become the norm.

Findings of the dietary change intervention reveal furthermore that if a sufficiently large subgroup of the model’s population converts to a vegan diet, the number of plant-based meals cooked substantially rises to meet the 50:50 goal. What is a sufficiently large subgroup, however, depends on the target group: high status individuals exert more influence than low status individuals. An essential nuance is that smaller-sized high-status groups can exert as much influence as larger-sized random groups. The effect of the random status group displays a more evenly distributed effect, though, implying that targeting consumers from all layers of Dutch society could have a more secure influence on transitioning towards more plant-based diets. Nonetheless, the association between status and normative behaviour remains a key component here. Although Chan and Zlatevska [72] found that eating meat provides status, in particular to those who experience a low social status, their finding does not include the relational aspect of status conferral and associated normative behaviour. If person A attributes a high status to person B preferring a vegan diet, it is very likely that person A will aspire to become a vegan, too, irrespective of the size of the vegan or non-vegan group.

### Limitations

In developing the MEET-eaters model we made several conceptual decisions that should be borne in mind when interpreting its results. First of all, a major limitation pertains to the fact that price was not considered in the cook-agents’ purchase decisions. Based on previous research, price appeared to be a complicated factor in the decision to purchase meat. While the relatively low price of meat compared to plant-based alternatives can serve as a barrier in reducing meat [73], other factors (e.g., status, hospitality, peer affiliation) indicate a less direct relationship between meat price and purchase [72,74–76]. A simulation study by Schenk *et al.* [77] reported “price” and “assortment” of grocery stores in northern Sweden as the most significant store attributes in the decision process of customers. Nonetheless, based on household-level market data, Briesch *et al.* [78], found that “assortments” are more important than “price” for consumer decision-making in a grocery store. These studies were limited, though, to aspects that are apparent inside a grocery store and left out any social influences that could mediate the effect of price and assortment. Secondly, once cook-agents are at the supermarket, they do not recall their dinner guests. Instead, the model simulates that cook-agents base their substitution choices on their level of neophobia. The study by Hesselberg *et al.* [79] found that upholding family harmony plays an essential role in the choice for reducing meat, implying that leaving out the effect of social influence on alternative purchases provides a limitation to our model. In addition, in MEET-eaters we assume that the cook does the groceries, while in real-life another household member may be responsible for this. A third limitation concerns the acquiring of meal ingredients. In the present model these are only purchased at physical supermarkets, excluding online grocery shopping and purchases from greengrocers, butchers, markets, convenience stores, or fast-food outlets. Excluding online grocery shopping may have led to an overestimation of cook-agents purchasing meat alternatives, similar to the effect of modelling supermarkets as offering only four protein source categories. Take-out and ready-to-eat meals are also not included, while these are typically consumed once a week by 38% and 48% of Dutch citizens, respectively [29]. A minor limitation pertains to the fact that during each dinner in MEET-eaters, only one type of meal is prepared, while within some household multiple are prepared. For instance, Veen *et al.* [80] report that commensality in student-only households with mixed dietary preferences (e.g., meat-eaters and vegetarians/vegans) is typically maintained by preparing meals that cater to everyone. Finally, person-agents in MEET-eaters adjust their dietary preference only based on mealtime experiences. Research addresses that communications through social media, including peer messaging, can potentially be another contributor to dietary change, although results are mixed [81–84]. Sudden crises, such as Bovine Spongiform Encephalopathy (BSE) disease, have been reported to decrease meat consumption, although only temporarily [85]. Thomopoulos *et al.* [22] simulated the effect of such crises in an agent-based model and found that these lead to the adoption of vegetarian protein sources. In addition, cooking skills were reported to influence the type of meat alternatives consumed at dinner among individuals seeking to reduce meat intake, as well as the perceived meal quality. In this context, ‘authentic’ meals were preferred over meat analogues, although this preference did not necessarily affect the decision to replace meat [14,86].

### Recommendations for future research

The MEET-eaters model is the first model that inquired the respective contribution of supply and demand to observed dietary patterns, explored mealtime negotiation processes, and tested the effect of intervening in food outlet product ranges and the dietary preference of the person-agent population, all in the context of attaining more plant-based diets. For future research, we recommend four expansions of the current model. First, to incorporate a stronger representation of the social and cultural dynamics responsible for the evolvement of practices, we recommend modelling with a more dynamic population and longer model runs. Doing so would require incorporating birth, death, and changes in household composition, as well as a social network informed by theoretical assumptions about dynamics in social networks and/or empirical data from existing networks, thereby providing a more realistic representation of the clustering of dietary identities. In particular, we recommend to include reference groups, any entity that is relevant to the individual, including friends, other vegetarians, the biosphere, or the self [27], to expand the dietary preference of person-agents to a concept influenced by social dynamics. Second, to include parent-child relationships, as sharing family meals is reported as the key factor in shaping children’s dietary habits, with social modelling and moderate restrictions (e.g., limiting unhealthy snacks) being the most influential parental behaviours [87]. Parents shape their children’s food environment through their food parenting practice (e.g., meals they provide) and their own dietary behaviour (e.g., social modelling) [88–90]. In addition, Costa and Oliveira [91] suggest that reciprocity in eating behaviour arises within the parent-child relationship, where children’s eating habits and parental feeding practices mutually influence each other. In particular avoiding conflict and upholding family harmony simultaneously serve as a driver and barrier to reducing meat consumption [79]. Third, include a more refined approach to select the cook. Since the cook in MEET-eaters is selected randomly, status or cooking skill does not affect the likelihood of preparing a meal, while those lacking culinary skills may become an unpopular choice for meal preparation. Gendered roles in cooking also pose a relevant factor to consider, as women are most often burdened with cooking and dinner choice [79]. Last, be more specific about the effect of masculinity and gendered roles on meat and veg*n consumption. From a biological perspective, men generally need more calories and also more protein compared to women because of their larger body size. Gender is found, however, to determine justification strategies for consuming meat [92]. Eating meat aids men in feeling masculine [93,94], yet men also may face social and cultural challenges, including emasculation, when reducing meat [95]. Historically, meat and masculinity are linked to social status, as those who were able to eat meat, were conferred more status by those who could not [72]. Within-gender differences are evolving, though, mediating men’s attachment to meat [96].

## Conclusion

MEET-eaters is the first agent-based model that enables exploration of the two-way interaction between supermarket supply and household demand and the role of decision-making within households about meals in a Dutch neighbourhood. In line with the increased understanding of dietary patterns as the emergent outcome of a complex system, results showed that both supermarket supply and household demand exert a substantial influence on the type of meals chosen. Results demonstrate that, since animal-based options are abundant in Dutch supermarkets, in particular meat, its high availability strengthens the practice of having meat for dinner. This study’s results demonstrate that targeting a population group with a large influence over others might be equally successful in shifting towards more plant-based diets than targeting any larger, random group. In addition, intervening with supermarket supply showed that plant-based meal choices were highest when animal-based options were restricted. Irrespective of the intervention duration, plant-based meal choices diminished after discontinuation of the intervention. As different approaches to choosing meals demonstrate a significant influence on type of meals chosen, more research is required on food decision-making processes within households. While current real-life interventions focus primarily on changing consumer behaviour, the findings of this study suggest that long-term, sustained policies targeting both supermarket supply and meal preference of particular consumer groups are most likely successful in the shift towards more plant-based diet.
